# Elaboration of Alumina-Zirconia Composites: Role of the Zirconia Content on the Microstructure and Mechanical Properties

**DOI:** 10.3390/ma6052090

**Published:** 2013-05-16

**Authors:** Valentina Naglieri, Paola Palmero, Laura Montanaro, Jérôme Chevalier

**Affiliations:** 1 Laboratorio di Tecnologia ed Ingegnerizzazione dei Materiali Ceramici (LINCE) Lab., Department of Applied Science and Technology, INSTM R.U. PoliTO, Politecnico di Torino, Corso Duca degli Abruzzi, 24, Torino 10129, Italy; E-Mails: valentina.naglieri@polito.it (V.N.); laura.montanaro@polito.it (L.M.); 2 Université de Lyon, INSA-Lyon, MATEIS UMR CNRS 5510, Bât. Blaise Pascal 7, Av. Jean Capelle, Villeurbanne 69621, France; E-Mail: jerome.chevalier@insa-lyon.fr; 3 Institut Universitaire de France, 103 bd Saint-Michel, Paris 75005, France

**Keywords:** Al_2_O_3_-ZrO_2_, nanocomposites, microstructure, mechanical properties

## Abstract

Alumina-zirconia (AZ) composites are attractive structural materials, which combine the high hardness and Young’s modulus of the alumina matrix with additional toughening effects, due to the zirconia dispersion. In this study, AZ composites containing different amounts of zirconia (in the range 5–20 vol %) were prepared by a wet chemical method, consisting on the surface coating of alumina powders by mixing them with zirconium salt aqueous solutions. After spray-drying, powders were calcined at 600 °C for 1 h. Green bodies were then prepared by two methods: uniaxial pressing of spray-dried granules and slip casting of slurries, obtained by re-dispersing the spray dried granulates. After pressureless sintering at 1500 °C for 1 h, the slip cast samples gave rise to fully dense materials, characterized by a quite homogeneous distribution of ZrO_2_ grains in the alumina matrix. The microstructure, phase composition, tetragonal to monoclinic transformation behavior and mechanical properties were investigated and are here discussed as a function of the ZrO_2_ content. The material containing 10 vol % ZrO_2_ presented a relevant hardness and exhibited the maximum value of *K_I0_*, mainly imputable to the t → m transformation at the crack tip.

## 1. Introduction

Zirconia toughened alumina (ZTA) is one of the most widely used composite oxide structural ceramics. In fact, for several years, ZTA composites have been used for wear parts and cutting tools, due to their excellent mechanical properties, such as high strength, hardness, toughness and abrasion resistance [1,2,3]. More recently, ZTA has become increasingly important as a structural material for biomedical implants, such as hip prosthesis. A key issue for such implants is to increase their lifetime, which is nowadays about 10 years. In fact, considering the increased life expectancy, as well as the growing demand of orthopedic surgery for younger and more active patients, implants should exhibit a lifetime of more than 30 years. For these reasons, research efforts currently focus on long-lasting devices based on new materials characterized by superior strength and toughness, optimal tribological properties and long-term biocompatibility [4].

In this frame, ZTA composites have demonstrated their effectiveness for orthopedic applications, and recently, the first composite femoral heads have been developed and commercialized. In this system, alumina provides high strength and hardness, whereas tetragonal zirconia exerts a toughening effect, thanks to its controlled transformation into the monoclinic phase [5,6]. In spite of the t → m transformation around advancing cracks having been recognized as the main toughening effect, other mechanisms can play a role, such as microcracking, crack deflection and bridging [7]. Microcracking is favored in ZTA with large un-stabilized zirconia inclusions, which become monoclinic during cooling. This leaves a network of microcracks in the alumina matrix, which enables high toughness, but limits strength [8]. On the other side, stress induced t → m transformation occurs in ZTA if the dispersion is kept tetragonal and transformable.

Generally, ZTA composites are prepared by the powder mixing route, whose main issue is keeping a homogeneous microstructure in the final, sintered materials. In fact, zirconia aggregates can lead to localized aging phenomena [9,10], whereas alumina ones could behave as preferential sites for crack propagation.

This work deals with the elaboration of ZTA composites through a simple and reliable method, which allows an effective tailoring of the powder characteristics, microstructure and properties of the fired bodies, as already demonstrated for other bi- [11,12,13,14] or even multi- [15,16,17] oxide composites. Such a route was here employed for preparing alumina-zirconia composite powders, in which the zirconia content ranged between 5 and 20 vol %. This work aims to demonstrate the key role of some processing parameters, such as the forming method, in producing fully dense sintered bodies with tailored microstructural features. The materials were submitted to a preliminary mechanical characterization, with the aim of correlating mechanical data to zirconia content and microstructural features.

## 2. Results and Discussion

**Figure 1:**
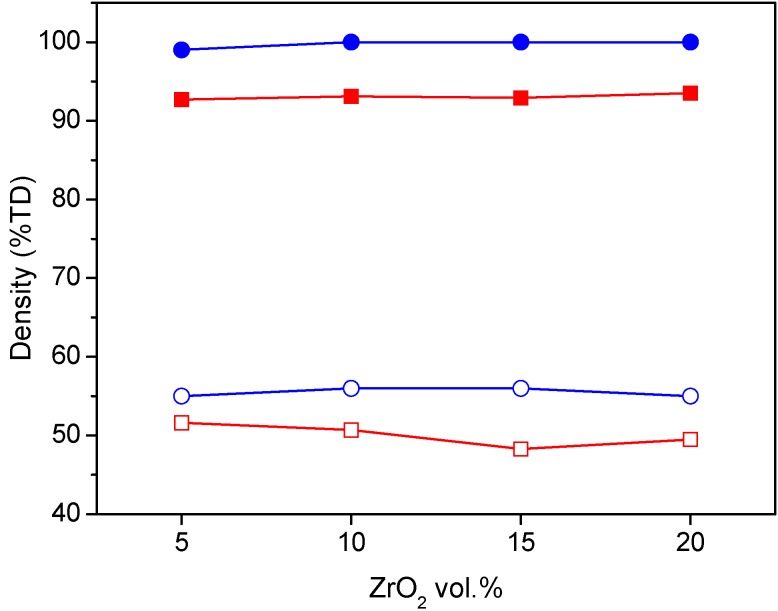
Green (empty symbols) and final (full symbols) density of alumina-zirconia (AZ) samples, obtained by uniaxial pressing (red) and slip casting (blue), as a function of the ZrO_2_ volume content.

AZ green bars, prepared by uniaxial pressing of the spray-dried powders, presented green densities in the range 48%–52% with respect to the theoretical density (TD), as shown in Figure 1. During sintering, carried out at 1500 °C for 1 h [18], all composites exhibited a similar densification behavior, in which the onset sintering temperature was at about 1100 °C and the maximum sintering rate temperature (as observed by the derivative curves) was about 1335 °C. After sintering, the materials reached relatively low final density, in the range 93%–94% TD.

**Figure 2:**
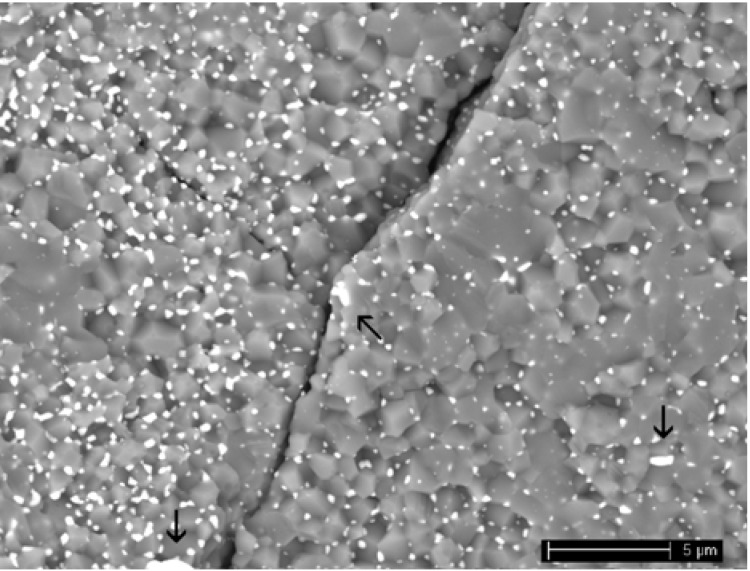
Low-magnification image of AZ5, obtained by uniaxial pressing of granulated powders.

By environmental scanning electron microscopy (ESEM) observation, residual pores were detected, in agreement with the density values. In addition, as shown in Figure 2 for AZ5, a very poor distribution of zirconia inside the alumina matrix was yielded in these composites, and in some cases, zirconia agglomerates were produced (see the arrows in Figure 2). Moreover, some large, macroscopic defects were also found in these materials, due to the un-optimized forming process.

**Figure 3:**
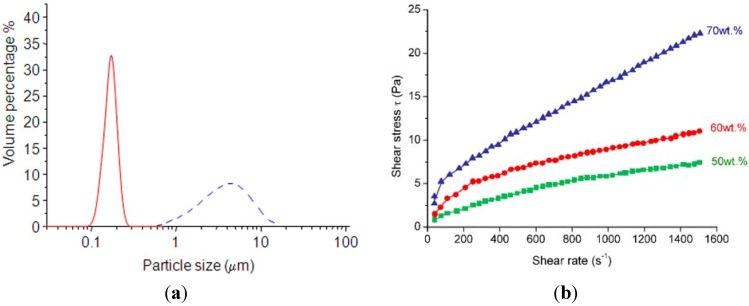
(**a**) Agglomerate size distribution of AZ5 before (blue, dashed line) and after (red, solid line) dispersion; (**b**) shear stress *vs.* shear rate for AZ5 suspension, as a function of the solid loading.

The reliability of fired materials was thus improved by slip casting, as reported in the following, which was performed with the aim of increasing the microstructural homogeneity in green and sintered bodies. All AZ aqueous suspensions were successfully dispersed by ball-milling: as an example, Figure 3a shows the particle size distribution of AZ5 before (blue, dashed line) and after (red, solid line) 3 h of ball-milling, in the case of a slurry whose solid loading was 50 wt %. The dispersion process was highly effective in breaking the spray-dried granules; in fact, the mean particle size (d_50_) of the ball-milled slurry was about 0.17 μm, one order of magnitude lower than the d_50_ of the powder before milling and comparable with the alumina primary particle size. Similar results were obtained for suspensions with higher solid loadings, but the time required for dispersion increased by increasing it. In fact, the slurries whose solid loading was 60 and 70 wt % reached a particle size distribution similar to that of Figure 3a, but after 24 and 60 h, respectively.

Figure 3b collects the shear stress values (τ) as a function of the shear rate [19] for the dispersed AZ5 suspensions with three different solid loading (50, 60 and 70 wt %); the yield stress (τ_Y_) and viscosity limit (η_Y_) of the same suspensions are collected in Table 1. Data show a relatively low viscosity for the suspensions at 50 and 60 wt %, whereas it significantly increased for a solid loading of 70 wt %.

**Table 1:** Yield stress (τ_Y_) and viscosity limit (η_∞_) for suspensions at different solid loadings.

Solid loading (wt %)	τ_Y_ [Pa]	η_∞_ [mPa^.^s]
50	0.3	3
60	0.7	4
70	1.7	7

**Figure 4:**
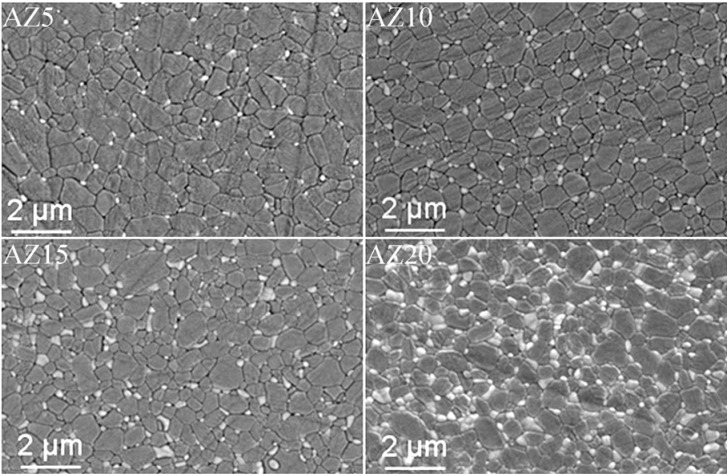
Environmental scanning electron microscopy (ESEM) images of sintered materials obtained by slip casting (observations on polished and thermally etched surfaces).

Thus, in order to reach the best compromise between high solid loading and low viscosity, AZ suspensions at 60 wt % were dispersed and cast. The slip cast green bodies were then sintered in the same conditions used for the pressed materials. The green and final density of the slip cast samples are collected in Figure 1. We can observe the effectiveness of such a wet-forming method in increasing both green and final densities, with respect to the pressed materials. All the slip cast samples reached full densification, independently from the zirconia volume content. Slip casting was also successful in increasing the microstructural homogeneity: in fact, a fully dense and highly homogeneous microstructure, in which very fine zirconia grains were well distributed inside the alumina matrix. In addition, the materials were defect-free, thanks to the proper powder dispersion and the improved quality of the green microstructure. In Figure 4, ESEM micrographs of the four composites are collected.

Image analysis, carried out on several ESEM micrographs, allowed to evaluate the alumina and zirconia size distribution. Their mean size, as a function of the ZrO_2_ content, is collected in Table 2. The zirconia mean grain size increased by increasing the ZrO_2_ content in the composites. On the opposite, the alumina grain size is inversely proportional to the zirconia content: the well-known *pinning* effect exerted by zirconia on the alumina grain size was effective in limiting the matrix grain growth, as already stated in the literature. For instance, Lange [20] reported that an optimum grain growth control can be achieved when the majority of the four-grain junctions contains ZrO_2_ inclusions, thus avoiding alumina abnormal grain growth.

**Table 2:** Alumina and zirconia mean grain size, as obtained by image analysis.

Sample	Al_2_O_3_ mean size (μm)	ZrO_2_ mean size (μm)
AZ5	0.88	0.26
AZ10	0.81	0.31
AZ15	0.75	0.31
AZ20	0.70	0.36

**Figure 5:**
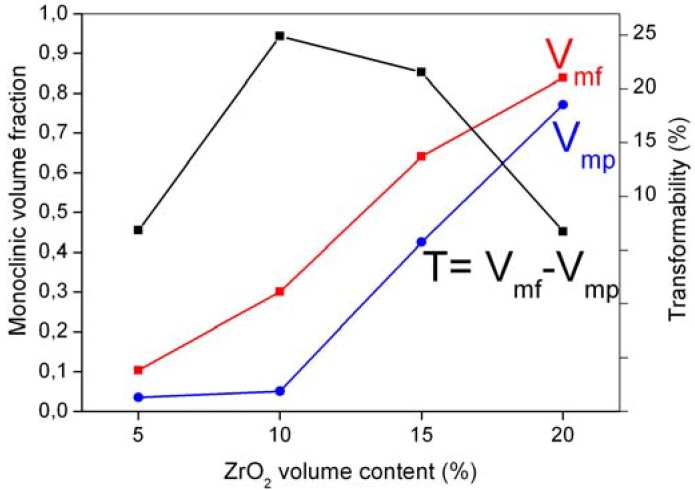
Monoclinic zirconia content in the polished (V_mp_) and fracture (V_mf_) surfaces and zirconia transformability, as a function of the ZrO_2_ volume fraction in the composites.

Figure 5 collects the monoclinic volume content, as determined by XRD analysis carried out on the polished (V_mp_) and fracture (V_mf_) surfaces [21]. In spite of V_mp_ being quite low for AZ5 and AZ10 samples (0.035 and 0.05, respectively), it significantly increased in the ZrO_2_-reacher compositions.

The conditions under which the tetragonal zirconia phase can be retained at low temperature have been the object of numerous papers, dealing with the thermodynamics of the t → m transformation in different systems, including ZTA composites [8,22,23,24]. In this two-phase material, the retention of tetragonal zirconia at room temperature depends on the elastic properties of the matrix: as Al_2_O_3_ is characterized by an elastic modulus, which is quite double that of ZrO_2_ [8], its stiffness is able to suppress the t → m transformation during processing. Moreover, in ZTA composites, residual stresses are normally present, due to the thermal expansion mismatch between the matrix and reinforcing phase. As the thermal expansion coefficient of alumina is lower than the tetragonal zirconia one [25], zirconia grains are subjected to a residual tensile stress, which promotes the transformation. The extent of such residual thermal stress depends on the zirconia content inside the matrix; precisely, it is inversely proportional to the zirconia volume fraction in the composites, as demonstrated by Becher *et al.* [26]. Finally, the tetragonal phase stability depends on the zirconia “critical” size, hereafter referred to as *D_C_*, above which spontaneous transformation can occur during cooling from sintering to room temperature.

**Figure 6:**
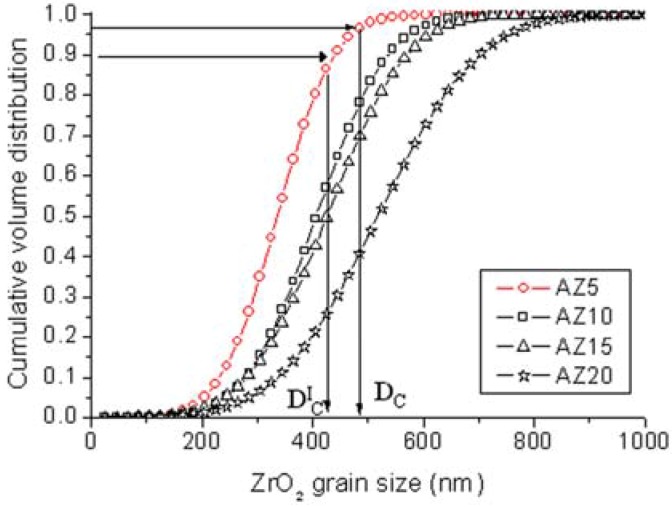
Cumulative volume distribution of ZrO_2_ grain size in the four composites; the estimation of *D^I^_C_* and *D_C_* is reported, as an example, for AZ5.

In this work, *D_C_* was evaluated by combining the cumulative size distribution of zirconia, shown in Figure 6, with the related amounts of transformed zirconia, as determined by XRD (Figure 5). In fact, as the transformability of zirconia grains increases by increasing their dimensions, it is assumed that all the transformed monoclinic particles are above such a critical size. The results are collected in Table 3 for the four AZ composites.

**Table 3:** Lower (*D^I^_C_*) and upper (*D_C_*) critical sizes of zirconia for the AZ composites.

Sample	D^I^_C_ (nm)	D_C_ (nm)
AZ5	435	485
AZ10	465	565
AZ15	385	445
AZ20	380	420

It can be observed that AZ10 critical size was larger than those of all other composites. For explaining this behavior, we should consider the opposite effects on the t-ZrO_2_ transformability played by the matrix constrain and residual stresses. In fact, the matrix constrain, which counteracts the t → m transformation, decreases by increasing the zirconia content. On the opposite, the tensile residual stresses, which promote the transformation, are more effective in alumina-richer compositions, as shown by Becher *et al.* [26]. According to this work, below the percolation limit (about 15 vol % of ZrO_2_), the influence of the residual stress prevails, so that the *D_C_* value obtained for AZ10 is greater than the AZ5 one. From 15 vol % ZrO_2_, the decreased influence of the matrix stiffness causes an increase in the zirconia transformability, and thus, *D_C_* decreases again.

As shown in Figure 5, V_mf_ also increased by increasing the zirconia content. The zirconia transformability, determined as the difference between V_mf_ and V_mp_, is depicted in the same Figure, showing a maximum value for AZ10. In order to deepen the dependence of the stress-induced transformation on the zirconia grain size, the lower critical size under which such transformation does not occur, *D^I^_C_*, was also evaluated. By following the same previous approach, *D^I^_C_* was estimated by coupling the V_mf_ data with the cumulative volume distribution of ZrO_2_ in the AZ composites, depicted in Figure 6. Results are collected in Table 3. The highest ZrO_2_ amount available for stress-induced transformation is reached when its dimension is in the range *D^I^_C_*–*D_C_*. The slightly larger *D^I^_C_*–*D_C_* range determined for AZ10 can explain its higher transformability, as compared to the other composites.

**Figure 7:**
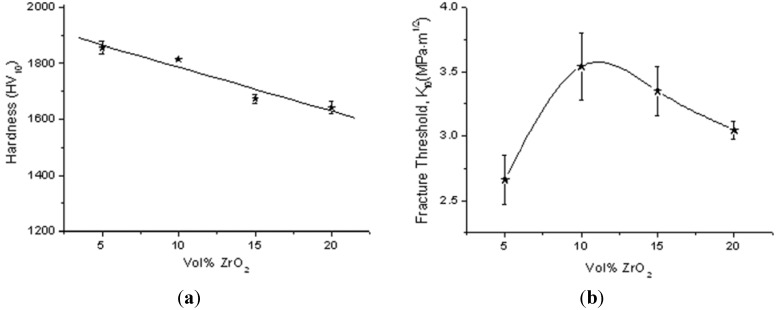
(**a**) Vickers hardness; (**b**) threshold for slow crack propagation obtained by the indentation test, as a function of the ZrO_2_ volume fraction in the composites.

Figure 7a collects the Vickers Hardness (*HV10*) as a function of the zirconia volume content. In spite of the decreased alumina grain size in the ZrO_2_-reacher composites, the hardness decreased from AZ5 to AZ20, as expected on the grounds of the rule of mixture [27]. The length of the indentation cracks were also measured, in order to evaluate the threshold for slow crack propagation. In fact, the radial cracks originated from the indentation grown under the driving force, due to the residual stresses, introduced by applying the load during measurements. Figure 7b depicts the fracture threshold, *K_I0_*, as a function of the zirconia content, showing a maximum for AZ10. This result is in agreement with previous literature data: in fact, a maximum is frequently observed in *K_C_* as a function of the zirconia content [5,7,9] in ZTA materials, containing un-stabilized zirconia. Generally, the toughness starts to decrease in correspondence to the ZrO_2_ concentration for which a substantial increase in monoclinic content is observed after sintering, in agreement with data shown in Figure 5.

The obtained *K_I0_* values, which are in good agreement with literature data [27,28], can be briefly discussed on the grounds of the two main toughening mechanisms active in ZTA composites, *i.e.*, microcracking and stress-induced t → m transformation. Microcracking is possible when the size of the ZrO_2_ inclusion exceeds a critical size (*D_s_*), which can be calculated according to the following Equation [13]:

(1) $$D_{s}=\frac{10\gamma_{m}}{{\left(\Delta \varepsilon \right)}^{2}\cdot E}$$

where γ_m_ is the fracture energy for microcracking (~1 J/m^2^), E the elastic modulus of the composite (~380 GPa, as determined by the rule of mixtures) and Δε = Δα·ΔT the product of Coefficient of Thermal Expansion (CTE) and sintering temperature difference [13]. From this calculation, a critical size of 1.35 μm was determined, significantly higher than the measured ZrO_2_ particles size in all the investigated AZ composites. Thus, a contribution to toughness from microcracking due to large, transformed zirconia particles was not expected. So, the higher (and similar) *K_I0_* values determined for AZ10 and AZ15 can be explained taking into account their higher zirconia transformability (Figure 5), thus strengthening the key role played by the stress induced t → m transformation over other toughening mechanisms.

**Figure 8:**
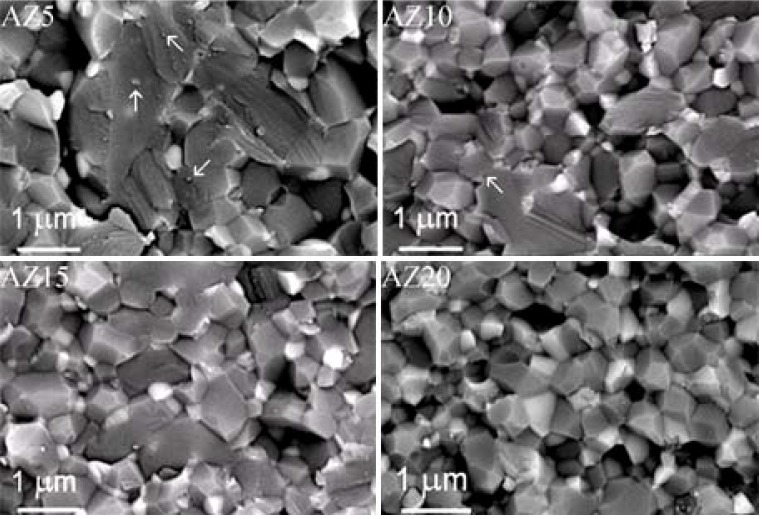
ESEM images of the fracture surface of AZ sintered materials obtained by slip casting.

Finally, the fracture surfaces of materials have been observed by ESEM, as given in Figure 8. Some different microstructural features can be observed. In fact, in AZ5 and AZ10, both inter- and trans-granular fracture modes seem to be present, as shown by the arrows, probably due to very fine ZrO_2_ particles located in the intra-granular position. In AZ15 and AZ20, the predominant fracture mode is the intergranular one, due to slightly larger zirconia grains, predominantly located along alumina grain boundaries and triple junctions. From this preliminary mechanical characterization, AZ10 was the most promising composition, and it was thus selected for developing femoral head prototypes [29]: this work is still in progress, and the results will be the object of a following publication.

## 3. Experimental Section

Alumina-ZrO_2_ (AZ) composite powders were prepared starting from commercial α-alumina powders (TM-DAR, Taimicron, Japan, d_50_ = 150 nm, S.S.A._BET_ = 14.5 m^2^/g). Well dispersed alumina suspensions were mixed with aqueous solutions of zirconium chloride. The doped slurries were maintained under magnetic stirring for 2 h and then spray dried. The powder was calcined at 600 °C for 1 h, to decompose by-products (mainly chlorides) and to induce the crystallization of tetragonal zirconia on the alumina surface. The elaboration method of the composite powders have been widely described in some previous papers [13,14]. The same procedure was employed to develop composite powders containing 5, 10, 15 and 20 vol % ZrO_2_; samples are hereafter referred to as AZ5, AZ10, AZ15 and AZ20, respectively. The volume percentage of zirconia into the composite powders was calculated assuming that it was fully tetragonal (6.05 g/cm^3^) in the final composites.

Green bodies were prepared by using two forming technologies. In the first case, the spray-dried granulates, calcined at 600 °C, were uniaxially pressed at 150 MPa. In the other, aqueous slurries, with solid loading ranging between 50 and 70 wt % were first dispersed by ball-milling, by using 3Y-TZP spheres. The particle size evolution, as a function of the dispersion time, was followed by laser granulometer (Malvern Masterzier 2000). The rheological behavior of each suspension was also investigated by using a Coulette rheometer (HAAKE VT 501, Villebon, France), at the following conditions: the shear rate (γ) was increased from 0 to 1500 s^−1^ in 2 min; the suspension was kept at the maximum shear rate for 1 min and then decreased to 0 once again in 2 min. The shear stress (τ) was thus measured according to the Casson equation [19]:

(2) $$\tau^{1/2}=\;{\tau_{\text{Y}}}^{1/2}+\;{\eta_{\text{∞}}}^{1/2}\;\gamma^{1/2}$$

where τ_Y_ is the yield stress and η_∞_ is the viscosity limit for infinite shear rate.

After that, the dispersed slurries were de-aired under vacuum for a few minutes and then cast into pure alumina porous molds and dried in controlled humidity atmosphere for 48 h. The samples were de-molded and left in static air for an additional 24 h.

The green bodies were submitted to dilatometric analysis, under the following sintering cycle [18]: heating rate of 10 °C/min up to 1100 °C, 2 °C/min up to 1500 °C, 1 h of holding time at the maximum temperature and a cooling rate of 10 °C/min to room temperature.

The as-sintered samples were submitted to density measurements using the Archimedes’ method. The theoretical density (TD) was calculated for each composition, assuming values of 3.98 and 6.05 g/cm^3^ for α-Al_2_O_3_ and tetragonal ZrO_2_, respectively. Sintered samples were submitted to X-ray diffraction (XRD) by using a Philips PW 1710 apparatus, operating with Cu Ka radiation (1.541, 874 Å); spectra were acquired in the range 5°–70° 2θ, with a step size of 0.05° 2θ and an acquisition time per step of 5 s. Diffraction patterns were indexed by using the Powder Data File database (P.D.F. 2000, International Centre of Diffraction Data, Pennsylvania). XRD were carried out on both polished and fractured surfaces; the intensities of the monoclinic (−111) and (111) reflexes, as well as the tetragonal (101) reflex were integrated, and the volumetric content of the monoclinic phase was calculated with the calibration curve of Toraya *et al.* [21].

The microstructural characterization of the sintered bodies was carried out by environmental scanning electron microscopy (ESEM, FEI XL30, Eindhoven, Netherlands) on polished and thermally etched surfaces, as well as on untreated fractured ones. Polishing was performed down to 1 μm with a diamond paste, whereas thermal etching was carried out at 100 °C below the sintering temperature, for 6 min.

The Vickers hardness was determined on all the samples by applying a maximum load of 98.1 N, by using a micro-indenter Vickers Testwell FV-700, equipped with an optical microscope. For each indentation, the length of the two diagonals was measured, and the corresponding hardness was determined according to standard equations [30]. The mean value of the Vickers hardness was calculated using data from at least 10 indentations. The Vickers indentation test was used to estimate the threshold for slow crack propagation, according to the Anstis’ formula [31]:

(3) $$K_{I0}=\chi \;P\cdot c^{-3/2}$$

where *P* is the applied load, *c* is the crack length measured from the center of indentation to the end of the crack and *χ* is a constant equal to:

(4) $$\chi \;=\;\zeta \;{\left(E/H\right)}^{1/2}$$

*ζ* being a material-independent constant, equal to 0.016, *E* the Young modulus and *H* the hardness. The Young modulus of the composites was evaluated by the rule of mixtures, assuming values of 400 GPa and 200 GPa for alumina and zirconia, respectively [8].

## 4. Conclusions

In this work, Alumina-Zirconia composite powders have been successfully produced by surface modification of a commercial alumina with aqueous solutions of zirconium chloride. This method allowed us to produce composite powders with the desired alumina-zirconia ratio, as well as with strictly tailored microstructural features, such as zirconia grain size and distribution. Composite powders with four different compositions were prepared, containing respectively 5, 10, 15 and 20 vol % of un-stabilized zirconia. Green bodies were prepared by two forming techniques, uniaxial pressing and slip casting. The second route was very effective in producing homogeneous and defect-free green bodies, which gave rise, after sintering at 1500 °C for 1 h, to fully dense microstructures, characterized by highly homogeneous distribution of ZrO_2_ particles inside the alumina matrix. The four composite materials have been compared in terms of different microstructural features, phase composition and mechanical properties. The composite containing 10 vol % ZrO_2_ exhibited good hardness and the maximum fracture threshold, *K_I0_*, which was mainly imputed to the t → m transformation at the crack tip.
